# The Role of *Cutibacterium acnes* in the Etiopathogenesis of Sarcoidosis: Current Insights and Future Study Directions

**DOI:** 10.3390/ijms26146652

**Published:** 2025-07-11

**Authors:** Angela Maria Di Francesco, Giuliana Pasciuto, Elena Verrecchia, Ludovico Luca Sicignano, Laura Gerardino, Donato Rigante, Raffaele Manna

**Affiliations:** 1 Periodic Fever and Rare Diseases Research Centre, Catholic University of Sacred Heart, 00168 Rome, Italy; angelamaria.difrancesco@unicatt.it (A.M.D.F.); donato.rigante@unicatt.it (D.R.); 2 Complex Pneumology Operational Unit, Fondazione Policlinico Universitario A. Gemelli IRCCS, Largo A. Gemelli 8, 00168 Rome, Italy; giuliana.pasciuto@policlinicogemelli.it; 3 Department of Aging, Orthopaedic and Rheumatological Sciences, Fondazione Policlinico Universitario A. Gemelli IRCCS, Largo A. Gemelli 8, 00168 Rome, Italy; elena.verrecchia@policlinicogemelli.it (E.V.); ludovicoluca.sicignano@policlinicogemelli.it (L.L.S.); laura.gerardino@policlinicogemelli.it (L.G.); 4 Department of Life Sciences and Public Health, Fondazione Policlinico Universitario A. Gemelli IRCCS, Largo A. Gemelli 8, 00168 Rome, Italy

**Keywords:** *Cutibacterium acnes*, sarcoidosis, granulomatous inflammation, innovative biotechnologies, personalized medicine

## Abstract

*Cutibacterium acnes* (*C. acnes*) is a commensal bacterium of the skin microbiota that can transform itself into a pathogen depending on the peculiar susceptibility of the host: it is the sole microorganism so far to be found in the specific organ lesions of sarcoidosis, and *C. acnes*-induced activation of T-helper-type-1 cell responses is generally higher in patients with sarcoidosis than in healthy subjects. This bacterium acts as an opportunistic agent in several inflammatory conditions other than sarcoidosis, such as prostate cancer and prosthetic joint infections. Both innate and adaptive immunity systems are involved in the pathogenesis of *C. acnes*-mediated sarcoid lesions, and a seminal role is played by host toll-like receptor (TLR)-2, TLR-4, TLR-6, NOD-like receptors, and mononuclear cell cytoplasmic receptors. This review summarizes current knowledge on the potential cause–effect relationship existing between *C. acnes* and sarcoidosis, addressing issues of future research directions and novel therapeutic strategies in the management of a complex disease such as sarcoidosis.

## 1. Introduction

Sarcoidosis is a complex systemic disease defined by the development of granulomas in a host of organs, predominantly affecting the lungs, lymph nodes, skin, eyes, central nervous system, and heart; moreover, it remains an insidious disease since the etiology is not fully elucidated yet and because the standard corticosteroid therapy may cause severe collateral effects [1]. The immune system is engaged in the *initiation* of an inflammatory response to different environmental triggers, leading to the formation of granulomas, the histologic hallmark of sarcoidosis; therefore, understanding its pathogenesis remains an important task to achieve in order to explore alternative therapeutic strategies [1].

Specifically, this mini-review deals with the relationship between *Cutibacterium acnes* (*C. acnes*) and sarcoidosis: the medical literature has been screened with the keywords “*C. acnes*”, “sarcoidosis”, “pathogenesis of sarcoidosis”, and the most significant papers have been selected as the main sources to highlight and review the updated know-how on this topic.

The aim is to summarize the present knowledge about the potential cause–effect relationship existing between *C. acnes* and sarcoidosis, clarifying the role of specific molecular patterns of *C. acnes* able to activate immunological pathways relevant in the pathogenesis of sarcoidosis. This will allow for future research and the development of novel therapeutic strategies in the management of the complex disease sarcoidosis.

## 2. *Cutibacterium acnes*: Commensal Bacterium and Opportunistic Pathogen

The genus *Cutibacterium* is a cluster of cutaneous microorganisms previously designated as *Propionibacterium*—which has been reclassified into four genera [2]—and is a Gram-positive bacterium considered as commensal; it represents a major component of the microbiota in human skin and eyes, though it is also well-represented in the anaerobic flora of both the intestinal tract and human gingival plaques (Table 1).

In particular, *C. acnes* is an ubiquitous Gram-positive anaerobe, slow-growing microorganism present in the sebaceous glands (on the face, back, and anterior chest region), with individual-specific rather than site-specific distribution [6]. *C. acnes* is usually considered a commensal, i.e., a member of the skin microbiota, established through adaptive immune tolerance mechanisms developed since the early neonatal period [7]. The epidermis has a complex structure with many functions, such as to protect and defend the body from external hazards by acting as a physical and immunological barrier, modulating the microbiota [8]. Healthy skin hosts microorganisms from multiple kingdoms: bacteria, fungi, and viruses; in particular, *Cutibacterium* biofilm formation is significantly augmented in the presence of staphylococci, enabling a robust proliferation under both anaerobiosis and aerobiosis [9].

*C. acnes* strains can be divided into the major types IA, IB, II, and III, according to sequence comparison of the *recA* or *tly* genes [10]. Recently, further discrimination has been provided by multi-locus sequence typing schemes and repetitive-sequence-based polymerase chain reaction protocols [11,12,13,14]. More specifically, *C. acnes* subtype I, also termed I-1a, is predominantly associated with moderate and severe forms of acne [12,13]. Conversely, *C. acnes* type II is largely reported as the most prevalent type in prostatic specimens from patients with prostate cancer (PCa) [15]. 

**Figure 1:**
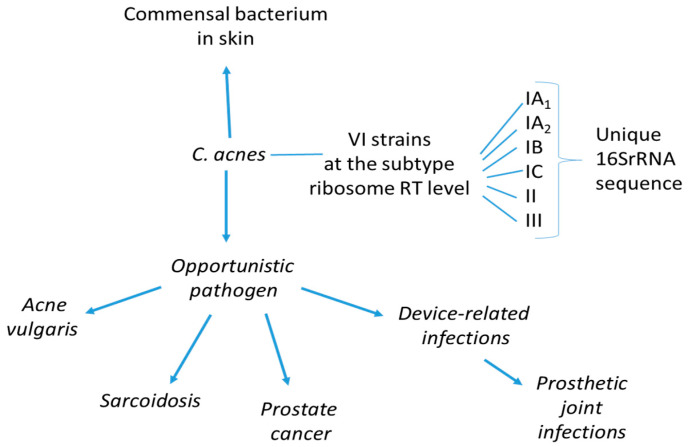
*Cutibacterium acnes* is considered an opportunistic microorganism, with the potential to switch from being a commensal to a pathogen causing different diseases. The *C. acnes* different phylotypes recognized by matrix-assisted laser desorption/ionization mass spectrometry prototyping are shown [21]. Each 16S rRNA sequence was defined as a ribotype (RT).

Furthermore, *C. acnes* is believed to play a remarkable role in maintaining skin health along ecological niches that could be colonized by more aggressive microbes via the production of short-chain fatty acids, thiopeptides, bacteriocins, and other compounds with inhibitory molecules against such organisms [16]. It also plays a role in the balance between healthy and inflamed skin (as in the case of acne disease) but may work as an opportunistic pathogen in other inflammatory conditions, including sarcoidosis, PCa, and prosthetic joint infections (Figure 1).

There is evidence of some disease-associated phylotypes of the bacterium persisting on body implants and causing postoperative inflammation, such as endocarditis, endophthalmitis, and central nervous system infections [17]. Tissue invasion and stockpile of *C. acnes* have also been reported in glandular epithelial cells, and circulating macrophages contribute to benign prostate hyperplasia [18]. Moreover, the prevalence of the phylotype IA-1 over the others, rather than a change in the abundance of *C. acnes*, is responsible for the development of acne [19,20].

However, beyond the evidence that these infections have involved different phylotypes of the bacterium, it remains important to ascertain whether the isolation of *C. acnes* strains should be considered as a true infection or a contamination of different tissues [3]. To date, it has not been established which biologic mechanisms are set in motion by *C. acnes* strains to result in infection, inflammation, and/or localization in distant parts of the body. However, it is known that bacteria-infected cells secrete pro-inflammatory cytokines and chemokines as well as anti-microbial factors, all of which play a role in the delicate balance between functional and dysfunctional conditions, such as a disease [22].

### 2.1. Molecular Markers of Cutibacterium acnes

*C. acnes* strains show both high- and low-inflammatory potential, although the individual colonization by a microorganism sequence does not predict host susceptibility to the disease [23]. It is largely accepted that almost all *C. acnes* subspecies and phylotypes share a similar invasiveness ability [24]. Several molecular markers exist that allow for the identification and typing of *C. acnes*, as well as characterizing its different pathogenic roles in many clinical conditions. For instance, lipidomic analysis helped in identifying specific lipid markers of *C. acnes* species such as phosphatidylcholine (PC) 30:0, sphingomyelins (SM) 33:1 and 35:1, derived phosphatidylglycerol (PG) with an alkyl ether substituent PG O-32, and cardiolipins/fatty acid amides, specific to different phylotypes and displaying potential diagnostic value [23,25].

Indeed, each *C. acnes* type (IA1, IB, II, and III) exhibits either pathogenic or commensal potential. Type I strains abundantly produce lipases, proteinases, and hyaluronidases, while type IA1 has been isolated from acne-prone skin; hence, it is considered a virulent strain, prevalent in acne vulgaris [26]. As for *C. acnes* lipidome, individual lipid compounds are considered markers for a given phylotype; i.e., *C. acnes* DSM 16379 (type IB) has a significant amount of PC 30:0. In addition to their obvious structural role, these PCs presumably contribute to virulence, confirming the pathogenic power of type I strains [27]. On the other hand, type II and III *C. acnes* are deemed “healthy skin” microbiota species [28]. While some strains are capable of producing sphingolipids, they can also acquire them from a mammalian host; these sphingolipids can then be modified by bacterial enzymes to produce new lipids, which help to conceal microorganisms from the host’s defense apparatus [29]. Noteworthy is sphingomyelin SM 35:1, which is highly observed in the *C. acnes* strains PCM 2334 and DSM 13655, which are type II and III, respectively. This finding supports the hypothesis that modification of host sphingolipids may lead to commensalism or hostile interactions as tissue damage and disease [29].

*C. acnes* produces two variants of hyaluronate lyase (HYL): the IB/II variant has a higher activity and can completely degrade the hyaluronic acid (HA) present in type IB and II strains; the IA variant has a lower activity and can partially degrade HA present only in type IA strains [30]. This difference in expression between *C. acnes* strains may account for the variable tissue-invasion capability between phylotypes. Moreover, type AI strains are found mostly on the surface of the skin in acne, whereas type IB/II strains are more frequently associated with deep soft-tissue infections [31]. Therefore, HYL is considered to act as a virulence factor by facilitating the bacterial invasion within tissues and degrading the compounds of the upper layers of skin and its extracellular matrix, thereby promoting the spread of inflammation. In addition, the products of HA degradation performed by HYL may be used as nutrients by the bacterium but may also influence the extent of inflammation [32,33].

T-helper-type-1 (Th1) immune responses to *C. acnes* catalase (KAT) have been measured by interferon (IFN-γ) assay in the peripheral blood mononuclear cells from 12 sarcoidosis patients, 13 patients with other pneumonitis, and 11 healthy volunteers; the KAT protein provoked a significantly higher response in patients with sarcoidosis [34].

The *C. acnes*-related inflammation produces matrix metalloproteinase (MMP) activation, contributing to tissue remodeling; other markers include adhesion factors and pore-forming toxins, CMP1 to CMP5 [35], lipase-mediated free fatty acids, and coproporphyrin III. The latter contributes to the perifollicular inflammatory reaction by stimulating the expression of pro-inflammatory molecules, such as CXCL8/IL-8 and prostaglandin PGE2 from keratinocytes, inducing the aggregation of *Staphylococcus aureus* and formation of biofilms in the nose [4].

### 2.2. Innate and Acquired Immunity in the Pathogenicity of Cutibacterium acnes

**Figure 2:**
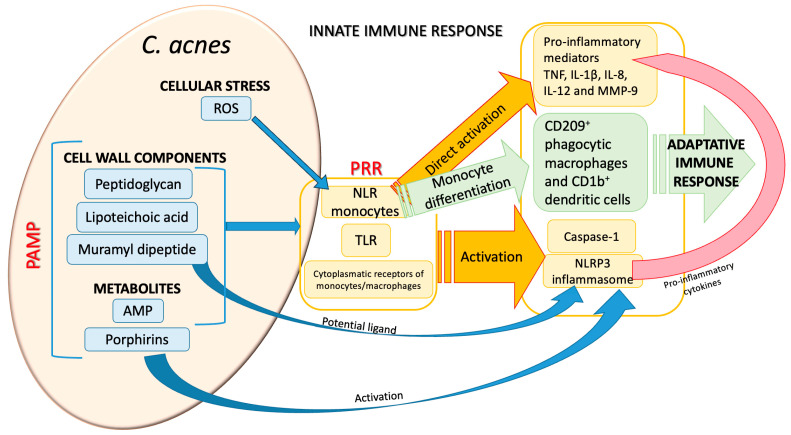
Major host recognition processes of *Cutibacterium acnes* and its pivotal innate immunity-mediated responses. ROS are highly triggering NLRs of monocytes, whereas muramyl dipeptide and porphyrins act directly on the NLRP3 inflammasome. PAMPs (pathogen-associated molecular patterns); PRRs (pattern-recognition receptors); NLRs (NOD-like receptors); NLRP3 (NLR pyrin domain-containing 3); TLRs (toll-like receptors); ROS (reactive-oxygen species); AMPs (anti-microbial peptides); TNF (tumor necrosis factor).

The causal relationship between a dysfunctional microbiome and disease states requires careful analysis, taking into account the heterogeneity of the bacterial strains, host genetics, and host environments. Many factors contribute to the development of *C. acnes* infection, first of all the role played by host immunity. As a commensal, *C. acnes* remains latent in the body until a mixture of overlapping triggering insults activates it, switching it to become pathogenic. In this process, *C. acnes* can elicit many different responses from the host: after the initial recognition process of the pathogen-associated molecular patterns (PAMPs) by the host pattern recognition receptors (PRRs), a cascade of chemokines is produced by the innate immunity system, followed by the host’s modulation of adaptive immune responses. The final effects of *C. acnes* on the host are the complex result of a fine balance between evasion and stimulation mechanisms. *C. acnes* also represents a priming agent, enhancing the efficacy of the host’s anti-pathogen response [36]. The first step in the switching process from a commensal to a pathogenic state implies the recognition process by the host. *C. acnes* has many arrows to its bow that allow it to be recognized by the host as a pro-stimulatory agent by its cell-wall components (peptidoglycan, lipoteichoic acid, muramyl dipeptide, etc.) or through metabolite production such as porphyrins [37] and anti-microbial peptides (AMPs) [38] (Figure 2). The recognition of *C. acnes* PAMPs is mediated by host toll-like receptors (TLRs) (TLR-2, -4, -6, and intracellular TLR-9 [35,39]), NOD-like receptors (NLRs), and monocytes/macrophages cytoplasmic receptors. NLRs can also be activated via reactive-oxygen species (ROS) released by *C. acnes*-stimulated cellular stress, as shown in *C. acnes*-mediated skin inflammation [40].

The PRR-mediated response results in the NLRP3 inflammasome, caspase-1 activation, and finally in an adaptive immune response. The inflammasome can also be directly activated by either muramyl dipeptide [41] or porphyrins [37], potentiating the *C. acnes* recognition process by the host. PRR activation elicits different signaling pathways leading to the production of pro-inflammatory mediators; activation of TLRs of monocytes produces tumor necrosis factor (TNF), IL-1β, IL-8, IL-12, and MMP-9 [42] and a further differentiation of these monocytes into different subsets, which in turn trigger the immune adaptive response [43]. Cytokine production stimulation by keratinocytes can be mediated either by viable bacteria [44] or *C. acnes* extracellular vesicles [45].

*C. acnes* can also evade the host response engaged to eliminate the pathogen attack, such as granuloma formation, extracellular traps, phagocytosis, autophagy, and pyroptosis, although little is known about the evasion mechanisms used by the bacterium [46].

### 2.3. Association of Cutibacterium acnes with Sarcoidosis

The isolation frequency of *C. acnes* was 78% in a group of 40 cases of sarcoidosis seen in a Japanese study, increasing to 92% when using high-osmolarity culture media [47,48]. Compared with sarcoidosis patients, the isolation frequency of *C. acnes* in biopsied lymph nodes from control patients without sarcoidosis was significantly lower (25% of 150 cases).

*C. acnes* can be found in the bronchoalveolar lavage (BAL) of approximately 70% of patients with sarcoidosis, where it is associated with disease activity, although it can also be found in 23% of controls [49,50]. Moreover, the immunohistochemistry approach has been used to detect *C. acnes* within the granuloma formation [51] by a commercially available *P. acnes*-specific monoclonal antibody (PAB antibody). Formalin-fixed paraffin-embedded tissue samples from 94 sarcoidosis patients and 30 control patients with other granulomatous diseases were examined by the original manual IHC method; *C. acnes* was detected in sarcoid granulomas of samples obtained by transbronchial lung biopsy (64%), video-associated thoracic surgery (67%), endobronchial ultrasound-guided transbronchial-needle aspiration (32%), lymph node biopsy (80%), and skin biopsy (80%) from sarcoidosis patients but not in any non-sarcoid granulomas of samples related to control subjects (with other granulomatous diseases). *C. acnes* signals were observed more frequently in immature granulomas compared to mature ones [52], suggesting that *C. acnes* may be degraded in the course of evolution of the granuloma. Therefore, sarcoidosis should be suspected when *C. acnes* is detected in granulomas, although sarcoidosis cannot be ruled out if *C. acnes* is not detected in granulomas.

**Figure 3:**
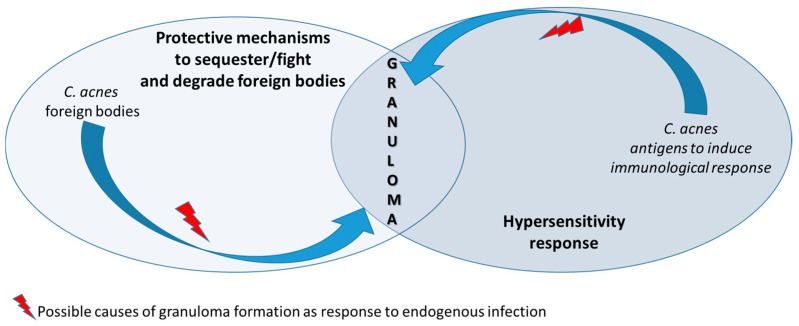
Granuloma represents a response to infectious microorganisms such as *Cutibacterium acnes* through overlapping of different responses. Sarcoid granulomas arise as a protective mechanism to counteract the spread of the infectious agent at sites of proliferating bacteria. Granulomas can be considered an expression of the innate immunity response in patients with hypersensitivity to *Cutibacterium acnes*.

Nowadays, molecular methods (Sanger sequencing of the 16S rRNA gene) in the mediastinal lymph node of patients with sarcoidosis have shown the presence of strains of *Streptococcus gordonii* (52 of 71 clones) and *C. acnes* (19 of 71 clones) [53]. Microorganisms may trigger the sarcoid reaction either because they act as antigens, inducing an immunological response, or by representing a non-degradable product [54] (Figure 3).

## 3. Sarcoidosis

Sarcoidosis is an intriguing disease studied for decades, and whose exact etiology remains elusive [55]. It would be useful to consider sarcoidosis as a syndrome comprising multiple genetic predisposing factors and many types of stimuli. Recent hypotheses converge on the idea that different environmental triggers (infectious, occupational, etc.) may induce a dysregulated inflammatory response in a genetically predisposed individual [25,56,57].

### 3.1. Genetics

The heritability of sarcoidosis may vary according to ethnicity. About 20% of African Americans with sarcoidosis have a family member with this condition, whereas for European Americans it is about 5%; additionally, in African Americans—who seem to experience a more severe and chronic form of the disease—siblings and parents of sarcoidosis cases have about a 2.5-fold increased risk for developing the disease [58]. In Swedish subjects, the heritability was found to be 39% [59]. In this group, if a first-degree member was affected, a person had a four-fold higher risk of becoming affected [59].

Investigations of genetic susceptibility have yielded many candidate genes, although few have been confirmed by further studies and no reliable genetic markers are currently known. The most interesting candidate gene is *BTNL2* [60], coding for butyrophilins that inhibit T-cell activation and act as negative costimulatory molecules in several conditions such as sarcoidosis, autoimmune diseases, and cancer. Several HLA-DR risk alleles have also been investigated. In persistent sarcoidosis, the HLA haplotype HLA-B7-DR15 is associated with the disease, either directly cooperating in disease development or via another gene between the two loci. In nonpersistent disease, a strong genetic association exists with HLA DR3-DQ2 [61]. Cardiac sarcoidosis (CS) has been connected to *TNF* variants, particularly in the gene promoter region, increasing CS severity. Specifically, certain haplotypes—including the A allele at position −308 and the T allele at position −857—have been found more prevalent in patients with CS [62]. There is an individual predisposition to develop *C. acnes* infection following a genetic pathway involving genes coding for innate immunity products, e.g., TLR-2, TLR-4, MAPK, NF-κB, IL-1, IL-6, IL-8, TNF, granulocyte–macrophage colony-stimulating factor from keratinocytes, and other inflammatory signaling pathways in the host [22,63]. As the NF-κB-dependent response to *C. acnes*, TLR-2 was shown to represent a critical receptor mediating the selective activation of innate immunity genes [35]. Therefore, the susceptibility of the host to a latent infection of the bacterium plays an important role in deciding the fate of the infectious process.

### 3.2. Immune Pathways

The hypothetical infectious triggers do not make sarcoidosis a simple infectious disease. Among the different microorganisms investigated, the only one that received confirmation was *C. acnes*, which has been isolated from sarcoid lesions by bacterial culture [47].

Invasive *C. acnes* can function as bacterial ligands to cause aberrant NOD receptor activation in predisposed individuals [64,65]. NOD1 and NOD2 are intracellular pattern recognition receptors that can sense bacterial molecules such as peptidoglycan moieties [66,67,68]. *C. acnes*-mediated aberrant NF-κB activation may induce granuloma formation in an NOD1/NOD2-dependent manner [22].

**Figure 4:**
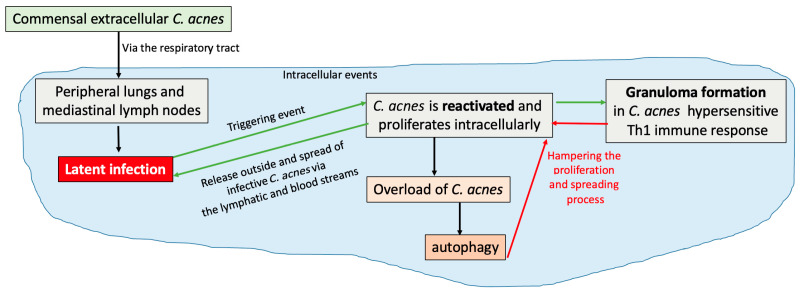
Proposed pathogenesis of sarcoidosis from a *Cutibacterium acnes* infection: intracellular events from latent infection to reactivation of the bacterium towards the formation of granuloma. Green arrows show pro-proliferation events, promoting spread of active infection around the body; red arrows show the potential mechanisms of *Cutibacterium acnes* containment and fighting that, in the absence of evasion mechanisms, can lead to infection resolution [54].

In recent years, evidence has suggested a role of T-helper-17 (Th17) cells in sarcoidosis. Researchers have found that IL-17A-expressing CD4+ T lymphocytes or IL-17A+IFN-γ+ memory T cells and RORγt, a nuclear receptor crucial for the differentiation of Th17 cells, are increased in both the BAL and the peripheral blood of patients with sarcoidosis [69,70]. IL-17A+ cells are also suggested to be persistently present in patients affected by sarcoidosis [69,70]. IL-17A+ cells are also suggested to be highly present in patients showing relapses of sarcoidosis [71] (Figure 4).

In the model of IL-17A-knockout C57BL/6 mice, the heat-killed *C. acnes* is able to induce sarcoidosis-like granulomas and even pulmonary fibrosis. In wild-type mice with granulomatosis, treatment with the anti-IL-17A antibody and administration of *C. acnes* enhanced IL-17A expression, together with granulomatosis and fibrosis, in mouse lungs after the boost stimulation. Neither granuloma nor fibrosis was observed in IL-17A-knockout mice, even in the presence of IFN-γ enhancement. Neutralizing the IL-17A antibody reduced inflammatory cells in the BAL and ameliorated both granulomatosis and fibrosis in mice with sarcoidosis. Therefore, IL-17A contributes to *C. acnes*-induced sarcoidosis-like inflammation in both granulomatosis inflammation and disease progression to pulmonary fibrosis [72].

The Th1-mediated response produces non-necrotizing granulomas that can localize anywhere in the body, making sarcoidosis a systemic disease [1]. The term “*C. acnes*-associated sarcoidosis” is applied to cases in which *C. acnes* is detected in granulomas via immunohistochemistry using the PAB antibody. This antibody reacts with a species-specific lipoteichoic acid of *C. acnes* in sarcoid granulomas and has been developed by mice immunization with the whole bacterial lysate, followed by immunohistochemical screening of *C. acnes*-specific antibody-producing hybridoma clones using formalin-fixed and paraffin-embedded samples of sarcoid lymph nodes [52]. PAB-antibody-positive Hamazaki–Wesenberg bodies (hypothesized to be cell-wall-deficient forms of *C. acnes*) were detected by these specific antibodies, mainly located the in sinus macrophages of lymph nodes; although non-specific for sarcoidosis, they can be detected at a higher frequency in sarcoid lesions as opposed to non-sarcoid ones (50% of 119 subjects versus 15% of 165 cases, respectively) [52]. Furthermore, the sarcoid specimens were shown to contain *C. acnes* DNA in different studies [73,74] and even in lymph node samples from European subjects recruited in an international study [67], supporting the hypothesis of a causative connection between the two conditions. Overall, sarcoidosis can be considered an endogenous hypersensitivity infection, which develops only after the following three factors are established: (1) a latent infection by *C. acnes*; (2) a reactivation of latent *C. acnes* triggered by environmental factors; (3) a hypersensitive Th1 immune response against the intracellular *C. acnes*. *C. acnes* is the sole microorganism ever isolated from sarcoid lesions, and activation of Th1 immune responses by *C. acnes* is higher in sarcoidosis patients than healthy subjects [34,75]. Some individuals with sarcoidosis have increased amounts of *C. acnes*-derived circulating immune complexes, which suggest a proliferation of this bacterium in multiple affected organs [76] (Figure 3). Indeed, current trials in subjects with CS are evaluating a combined treatment with corticosteroids and anti-microbials during active disease with continued anti-microbial therapy while tapering off steroids after the disease subsides [77].

### 3.3. Etiopathogenesis: What Role for Cutibacterium acnes?

*C. acnes* can be assumed to be one potential trigger of sarcoidosis. The pathogenesis of the process implies a first passage from being an extracellular commensal to an intracellular latent infectious agent in peripheral lungs and mediastinal lymph nodes [54]. The in vitro model of persistence of *C. acnes* in human blood can mimic in vivo conditions [78] in order to investigate the cellular processes during granuloma formation. In particular, the *C. acnes* strain isolated from prosthetic joint infection (PJI) induced a higher recruitment of CD8+ lymphocytes inside the granuloma. In patients suffering from *C. acnes* PJI, these lymphocytes, through their cytotoxic activity, may cause tissue damage leading to osteoclast activation and thereafter aseptic loosening of the prosthesis [79], which is frequently observed during chronic and low-grade infections due to *C. acnes* [80]. In contrast, in acne-related sarcoidosis, a high recruitment of CD4+ lymphocytes is in accordance with previous studies, demonstrating the main role played by this specific lymphocyte subset [81,82]. Interestingly, the *C. acnes* S8 strain recovered from the lymph node of a sarcoidosis subject was able to produce the highest number of granulomas; moreover, the bacterial burden inside the granulomas was significantly higher with this strain. Based on its clinical origin, it has been supposed that strain S8 harbored several antigens leading to the formation of granulomatous structures [83].

**Figure 5:**
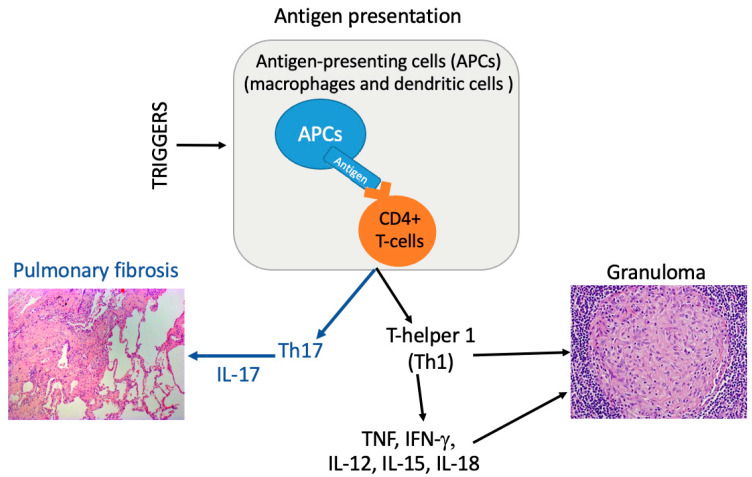
A synthetic schematic view of main immunopathogenesis pathways of sarcoidosis [85]. APCs: antigen-presenting cells; CD4+ T cell: cluster of differentiation 4 T cell; Th: helper T; INF-γ: interferon-gamma; TNF: tumor necrosis factor.

The granuloma formation can be affected by *C. acnes* KAT expression, as this enzyme induces hypersensitive Th1 immune responses in sarcoidosis [34,84]. It has been reported that granuloma formation takes place only in predisposed individuals hypersensitive to *C. acnes* through a Th1 immune response. *C. acnes* proliferating intracellularly can, therefore, be confined by the growing granuloma triggered by the bacterium and hampered by local autophagy processes, leading to a final resolution of the granulomatous inflammation and of the sarcoidosis process. The alternative fate occurs when *C. acnes* escapes either the granulomatous confinement or the other host defense strategies, leading to the spreading of intracellular latent infection from the respiratory tract to other organs of the body through bloodstream, where reactivation by triggering events can cause systemic sarcoidosis [54] (Figure 5).

## 4. Novel Therapeutic Options

*C. acnes*-related sarcoidosis has been treated with anti-microbial agents. There are several reports or small case studies in which antibiotic treatment resulted in improved clinical symptoms [86,87]. When minocycline was used, treatment discontinuation resulted in symptom relapse; therefore, as tetracyclines have anti-inflammatory properties, these results have been interpreted as a consequence of an immunomodulatory effect rather than a true anti-microbial effect of the treatment [86,88]. In the same direction is the case report of cutaneous sarcoidosis in a tattooed subject, showing improving symptoms after minocycline treatment [89]. However, the immunohistochemistry detection of *C. acnes* with a PAB antibody on cutaneous sarcoidosis of a subject undergoing permanent makeup raises the hypothesis that the anti-microbial action might play an important role in treating these *C. acnes*-related sarcoidosis subjects [90].

The Japanese Antibacterial Drug Management for CS (J-ACNES) trial showed interesting results of anti-microbial therapy *plus* corticosteroids compared to corticosteroid therapy alone in CS [77]. The standard treatment of CS is lifelong corticosteroid therapy with the need for dose escalation in order to prevent the inflammation from worsening, with dose-dependent adverse effects. The use of corticosteroid-sparing drugs such as methotrexate to contain adverse effects produced limited data so far [91], making the use of anti-microbials an interesting option to evaluate. The rationale for the J-ACNES trial was the identification of *C. acnes* in sarcoid granulomas of myocardial tissues of CS subjects [92], suggesting an etiologic role for *C. acnes*. The use of anti-microbial monotherapy was not effective against CS [93]. In the J-ACNES trial, a combination of anti-microbial drugs (clarithromycin 200–400 mg/day and doxycycline hydrochloride 100–200 mg/day) in addition to corticosteroids was used for 6 months and displayed a good safety profile, mimicking the clinical strategy of long-lasting drug combinations that are successfully used in other granulomatous diseases, such as tuberculosis and leprosy [94]. The results of this trial are still under investigation and it will be interesting to evaluate them in the light of sarcoidosis pathogenesis. The PHENOSAR trial (use of antibiotics in the treatment of sarcoidosis, NCT05291468) [95] was initiated in the Netherlands during 2022, although the study has passed its completion date, and its status has not been updated after two years. This trial represented the first study based on a targeted therapy rationale for sarcoidosis, i.e., the presence of *C. acnes* in the granulomatous tissues of sarcoidosis subjects. Two antibiotics were administered for 13 weeks, azithromycin and doxycycline, and the results were expected to be compared between placebo and treated groups; inflammation status was expected to be monitored by PET/CT scans and serum biomarkers —angiotensin-converting enzyme and the IL-2 receptor. Unfortunately, nothing is known so far from the study; therefore, the question of whether the presence of *C. acnes* in sarcoidosis patients might represent a successful strategy awaits further investigation.

## 5. *Cutibacterium acnes*, Sarcoidosis, and Malignant Tumors

Chintalapati et al. found that *C. acnes* can be isolated from tumors since the host response to the microorganism resulted in an ”immunologic hub” at the infected niches responsible for anti-tumoral activity [96]. *C. acnes* has been used as an immunostimulant adjuvant therapy for malignant tumors since the 1970s/1980s: indeed, when injected as a heat- or formol-inactivated suspension, this bacterium induced immunomodulatory effects on both innate and adaptive immune responses. Its anti-tumoral activity has been demonstrated in murine models [97]. *C. acnes* has also been used as a priming agent to enable host cells to respond efficiently to a pathogen attack: McCaskill et al. established an in vivo model of *C. acnes*-induced pulmonary inflammation, in which mice were intraperitoneally sensitized and intratracheally challenged with heat-killed *C. acnes* [36]. The study revealed a significant increase in leukocyte recruitment to the lungs and cytokine production compared to both controls and non-sensitized but challenged mice. This anti-tumor activity appears to be strain-specific, with certain *C. acnes* types exhibiting stronger effects than others. For example, *C. acnes* type I showed a higher survival rate in mice than type II [98]. Differences in the carbohydrate composition of cell walls of different *C. acnes* strains may contribute to their varying immunological and biological activities, potentially influencing their anti-tumor properties [99]. Some *C. acnes* strains not only inhibit tumor growth but also the spread and growth of metastasized tumor cells [100]. In fact, some clinical trials have reported the use of *C. acnes* in the treatment of various cancers (ovarian carcinoma, malignant melanoma, lung cancer, and breast cancer), while the disease-free survival rate was shown to be significantly increased in patients who received this adjuvant therapy [96]. Additionally, a recent study showed that tumor-isolated *C. acnes* activated the immune system, and immune cells effectively penetrated through the tumor tissue and formed an immunologic hub inside, explicitly targeting the tumor and destroying its malignant cells [101]. The anti-tumor activity was mainly attributed to local stimulation of lymphokine production (IL-12, IFN-γ, and TNF) resulting in T-cell recruitment, proliferation, and orientation towards a Th1 profile [101,102], as well as non-specific macrophage activation leading to tumor size reduction [103].

These data are consistent with a recent retrospective investigation [104] conducted on 287 sarcoidosis outpatients assessed between 2000 and 2024; diagnosis of cancer was recorded in 36 subjects (12.5%), and cancer preceded sarcoidosis or sarcoid-like disease in 63.8%, while sarcoidosis accompanying the onset of malignancy was 27.8%, and cancer arising after sarcoidosis diagnosis was only 8.3%. Only 2 out of 36 subjects with sarcoidosis and cancer showed metastasis, and one of them was affected by lymphoma. These data suggest that granulomatous inflammation due to sarcoidosis represents a protective shield, preventing the spread of metastasis through the induction of immune surveillance against cancer, while, on the other hand, it can be a risk factor for lymphomagenesis due to a persistent, chronically active inflammatory status [104].

## 6. Future Directions for Research and Treatment

A rational approach to the treatment of sarcoidosis should include precision in etiological and microbiological investigations, with the ancillary aim of avoiding infectious complications of either corticosteroids or immunosuppressors; therefore, the use of a combination of anti-microbial agents and immune regulators in *C. acnes*-related sarcoidosis subjects seems promising because of the clinical effects in limiting the use of corticosteroids. Indeed, it is a common practice in rheumatology to investigate the pre-existence of latent tuberculosis or persistent viral infections before starting steroid treatment or immunosuppression. Therefore, the coexistence or presence of *C. acnes* as a trigger for sarcoidosis should recommend a preventive treatment before steroid treatment or immunosuppression. Since the non-specific and multidrug therapies induce the development of resistant strains (such as rifampicin-resistant strains in leprosy treatment) [105], according to personalized medicine, the typing of acne strains and the use of specific antibiograms should be used. This procedure has not yet become routine, although it should be considered as a potentially fruitful clinical practice. Therefore, a combination of strategies that take into account both the etiology and pathogenesis could be the best approach to prevent and restrain the granulomatous manifestations observed in subjects with sarcoidosis.

## Figures and Tables

**Table 1:** Some of the major identity characteristics of the microorganism *Cutibacterium acnes* [2,3,4,5].

*Cutibacterium acnes*
Aerotolerant anaerobe
Non-spore-forming
Gram-positive
Rod bacterium, diphtheroid, or coryneform (slightly curved, with a width of 0.4–0.7 µm and a length of 3–5 µm)
Cell wall consists of phosphatidylinositol, triacylglycerol, other lipids, and peptidoglycan with L-acid, L-diaminopelic acid, and D-alanine in the peptide chain
It expresses the following proteins (for oxidative phosphorylation): NAPDH dehydrogenase/complex I, cytochrome c reductase, cytochrome c oxidase, and FoF1-type ATP synthase
Indigenous to skin and mucosal surfaces: it predominantly resides in the pilo-sebaceous follicle of the skin and also normally resides in the oral cavity, conjunctiva, external ear canal, and gut
Slow-growing (5-to-7 days with a division time of about five hours)
The genus comprises five species (*Cutibacterium acnes*, *Cutibacterium avidum*, *Cutibacterium granulosum*, *Cutibacterium namnetense*, and *Cutibacterium modestum*)

## Data Availability

Not applicable.

## Abbreviations

The following abbreviations are used in this manuscript:

AMP	Anti-microbial peptides
APC	Antigen-presenting cell
PAMPs	Pathogen-associated molecular patterns
HA	Hyaluronic acid
HYL	Hyaluronate lyase
INF-γ	Interferon-gamma
KAT	Catalase
NLR	NOD-like receptor
NLRP3	NLR pyrin domain-containing 3
PAB	*Propionibacterium acnes*-specific monoclonal antibody
PRRs	Pattern recognition receptors
PCa	Prostate cancer
PC	Phosphatidylcholine
PG	Prostaglandin
ROS	Reactive oxygen species
RT	Ribotype
SM	Sphingomyelin
TLR	Toll-like receptors
TNF	Tumor necrosis factor
